# Integrating electromagnetic cancer stress with immunotherapy: a therapeutic paradigm

**DOI:** 10.3389/fonc.2024.1417621

**Published:** 2024-08-06

**Authors:** Mark M. Fuster

**Affiliations:** ^1^ Research Service, VA San Diego Healthcare System, San Diego, CA, United States; ^2^ Pulmonary & Critical Care Division, University of California, San Diego, San Diego, CA, United States; ^3^ Department of Cellular & Molecular Medicine, Glycobiology Research and Training Center, University of California, San Diego, San Diego, CA, United States; ^4^ Veterans Medical Research Foundation, San Diego, CA, United States

**Keywords:** electromagnetic, cancer, pulsed, magnetic, glycocalyx, immunotherapy

## Abstract

An array of published cell-based and small animal studies have demonstrated a variety of exposures of cancer cells or experimental carcinomas to electromagnetic (EM) wave platforms that are non-ionizing and non-thermal. Overall effects appear to be inhibitory, inducing cancer cell stress or death as well as inhibition in tumor growth in experimental models. A variety of physical input variables, including discrete frequencies, amplitudes, and exposure times, have been tested, but drawing methodologic rationale and mechanistic conclusions across studies is challenging. Nevertheless, outputs such as tumor cytotoxicity, apoptosis, tumor membrane electroporation and leak, and reactive oxygen species generation are intriguing. Early EM platforms in humans employ pulsed electric fields applied either externally or using interventional tumor contact to induce tumor cell electroporation with stromal, vascular, and immunologic sparing. It is also possible that direct or external exposures to non-thermal EM waves or pulsed magnetic fields may generate electromotive forces to engage with unique tumor cell properties, including tumor glycocalyx to induce carcinoma membrane disruption and stress, providing novel avenues to augment tumor antigen release, cross-presentation by tumor-resident immune cells, and anti-tumor immunity. Integration with existing checkpoint inhibitor strategies to boost immunotherapeutic effects in carcinomas may also emerge as a broadly effective strategy, but little has been considered or tested in this area. Unlike the use of chemo/radiation and/or targeted therapies in cancer, EM platforms may allow for the survival of tumor-associated immunologic cells, including naïve and sensitized anti-tumor T cells. Moreover, EM-induced cancer cell stress and apoptosis may potentiate endogenous tumor antigen-specific anti-tumor immunity. Clinical studies examining a few of these combined EM-platform approaches are in their infancy, and a greater thrust in research (including basic, clinical, and translational work) in understanding how EM platforms may integrate with immunotherapy will be critical in driving advances in cancer outcomes under this promising combination.

## 1 Introduction to electromagnetism and cancer

A growing literature has demonstrated unique and sometimes striking effects of electromagnetic (EM) fields on tumor cells and animal experimental tumors (1–8). This includes exposures to some human and large-animal tumors under compassionate therapy platforms with encouraging responses (9–12) while signaling a need to examine these phenomena in rational studies guided by an improved understanding of mechanisms. A small array of EM “platforms” (13, 14) in the literature examine a scatter of physical variables, often reporting effects of a single frequency or amplitude on cells or tumor growth, with a range of exposure types that are difficult to compare. As such, it is difficult to extrapolate the effects of delivery variables other than frequency (Hz) or maximum amplitude (Tesla) of experimental radiofrequency EM waves or pulsed EM fields. Are there other critical variables to examine? What about the mechanism? Transduction of such stimuli to affect cell biological behavior or outcomes may occur through classical (e.g., Faraday induction) or non-classical (quantum spin) mechanisms, and studies to understand this in unique contexts are desperately needed (15–17). Even empirical studies that help one understand the most simple “dose responses” to the most relevant variables that impact cellular or whole-tissue functions (e.g., the viability of all or part of a tumor) would be highly valuable for rational research growth and clinical translation. To be clear in terms of exposure to an “EM platform”, this herein refers to the application of electromagnetic waves or pulsed electric or magnetic fields that are non-ionizing and non-thermal with regard to tissue interactions (whether applied externally or in direct contact with a tumor target).

Despite these challenges and fragmentary insights, an early common theme emerging from such research is that the overwhelming effect of exposing tumor cells or whole experimental tumors to EM platforms is a notable inhibition in tumor cell growth and augmented tumor apoptosis as a result of such exposures (3–5, 7, 18–20). There are, of course, many nuances to consider when an *in vivo* tumor is exposed to any given EM platform: these include direct tumor cell effects, stromal cell effects, immunologic impact, blood flow, the augmentation of parallel therapies (e.g., pairing EM exposure with chemotherapy or other treatments), or undefined effects on the whole organism. Nevertheless, when tumor cell lines are exposed *in vitro*, the overall effect of tumor-cell growth inhibition and apoptosis induction appears to hold. In the setting of carcinomas, this leaves a variety of questions, including the effects on heterogeneous carcinoma sub-clones that may have unique antigenic make-up or landscapes or the effects on tumor cells with distinct propensities to invade or metastasize. There may also be unique susceptibility of tumor mitochondria to the generation of reactive oxygen species or apoptosis induction (or their plasma membranes to electroporation phenomena). Cancer stem versus non-stem cells may also be differentially affected. All of these may depend on which EM platform is applied in any given exposure.

There is also a rationale for integrating anti-tumor immunity strategies with such EM platforms. Any number of the above exposures may lead to conditions that promote anti-cancer immunity, with an opportunity to apply such unique physical states to tumor cells as novel “substrates” for mechanisms that boost anti-tumor immunity. The paradigm presented herein examines how biophysical parameters of EM waves or pulse sequences driven by EM wave-generating or magnetic sources may modulate a cancer cell population and/or a tumor microenvironment (TME) to promote conditions that facilitate anti-tumor T-cell immunity and in some cases even humoral immunity, as it involves distinct classes of immune cells. A major focus is the optimization of states that promote the endogenous tumor-specific immune synapse (21). This includes augmented driving of immunologic signal-1 events (i.e., antigen-driven T-cell engagement as the “first signal”) to maintain anti-tumor immune specificity and efficacy in the setting of an unstable and pleomorphic carcinoma-cell landscape.

## 2 Electromagnetic fields and cancer cell engagement: physical variables

### 2.1 Electromagnetic waves and the cell: anatomic and functional considerations

How may one “engage” any given part of a cell with electromagnetic waves of any type? An electromagnetic wave is a traveling wave composed of electric (*E*) and magnetic (*B*) field vectors that are orthogonal to each other and classically travel through space in a direction perpendicular to the *E* and *B* vectors, with the power of the EM wave defined as the Poynting vector (cross-product of the maximum *E*- and *B*-field components).

Such waves have often been tested in the radiofrequency range, which can penetrate tissue to some extent (RF “skin depth”, limited by tissue dielectric properties) and, with enough power, can induce thermal effects. An electric field applied to a tissue surface generally attenuates greatly upon entry from atmospheric space, with greater tissue depth at lower frequencies (e.g., plane wave distances on the order of centimeters in muscle in the MHz–GHz range) (22). A variety of frequencies from the extremely low frequency (ELF; <300 Hz) to radiofrequency (kHz–MHz) range have been used to alter the growth and/or apoptosis of cultured tumor cells at energy levels that do not necessarily induce thermal effects, particularly with EM platforms used to generate pulsed magnetic fields at frequencies under 100 kHz (13, 23). At greater frequencies (GHz–THz), low energy (non-thermal) delivery modes can be applied, but tissue attenuation may be greater, and data do not necessarily reveal greater anti-tumor effects at higher frequencies (13, 24). Nevertheless, higher ranges (gamma or X-ray) result in significant ionizing potential. The focus herein is on non-thermal and non-ionizing EM platforms in an attempt to minimize tissue damage, including any harmful effects on bystander immune cells, including T cells that may be sensitized or recruited if naïve within a tumor microenvironment.

Independently, a magnetic field can be applied, either statically or in oscillating or pulsed formats (13). If the *B*-field is pulsed or oscillates, this can generate induced electromotive forces (EMFs) by Faraday’s law operating within the tissue through which the *B*-field fluxes (25). The induced EMF would then be able to drive or force any charged mass (e.g., anionic glycans in the tissue or tumor membranes) in the tissue. The flux operates across any theoretical conductor of charge deep in the tissue defined by the product of dB/dt (where the *B*-field changes from min to max over interval time “dt”) and the area (A) bounded by the “circuit” perimeter over which the induced potential/EMF operates (in this case, where the *B*-field moves perpendicular through area A bounded by the EMF induced along the perimeter of A). The oscillations or pulsations may be pulsed over any arbitrary “duty cycle”, even over a very narrow “rise time”, allowing for relatively low-magnitude *B*-fields to generate appreciable dB/dt over a reasonably small cross-sectional area, A, through which the field fluxes to generate the EMF along the perimeter of A.

Engaging such fields physically with cellular elements or characteristics that are unique to tumor cells becomes functionally interesting while ensuring that normal cells remain intact/unaffected. Targeting or physically engaging with a charged or ion-dense cancer cell-specific component with any EM-wave or *B*-field induction approach is a central interest, disrupting a tumor cellular structure or process that ultimately i) ablates the tumor cell or ii) leads to greater exposure of local immunity to tumor antigens. While these “cytotoxic” approaches would ideally fully spare normal surrounding stromal cells/tissues, one may consider approaches that achieve a greater probability or magnitude of detrimental effects (e.g., immediate or delayed cell death) in cancer cells than that of surrounding host cells. The latter may simply express a lesser degree of unique targets (e.g., charge density) that make the cancer cell vulnerable to the EM platform. Such features include charged elements critical for cell proliferation, energetics, or redox homeostasis (e.g., S-phase chromatin/DNA, microtubule elements, mitochondrial membrane, or downstream free-radical oxygen species) or charged glycans that are integral to the tumor membrane glycocalyx (e.g., glycosaminoglycans and/or sialic acid modified glycoproteins) (17, 24, 26–30). Electric conduction via a highly anionic tumor-membrane contiguous glycocalyx or tumor cell–cell junctions may permit lesser resistance for electric conduction of induced EMFs. At the quantum level, electron spin pairs may be uniquely susceptible to pulsed magnetic fields, which can impact biological phenomena at energies below the thermal background (17, 31). Further, pulsed electric field (PEF) delivery probes may be used to contact a tumor border directly, inducing membrane stress and even cancer-cell pore induction while taking advantage of unique tumor-conducting properties. Electroporation of tumor cells with its associated stress and tumor-cell death is a major mechanism in such applications (32).

Apoptotic and growth-inhibitory tumor cell responses appear to evolve secondary to many of these perturbations (13, 23). Potentially, a variety of “upstream” impacts from EM field exposures could evolve to drive apoptosis, including EM-driven cell membrane nanopore formation and associated cell stress, integrin-associated downstream focal adhesion and cytoskeletal signaling alterations, reactive oxygen species (ROS) generation with mitochondrial-driven apoptotic pathway activation, and possibly other pathways involving microtubular effects or effects on cyclin-dependent kinases that impact cell division (13, 24, 30). In addition to apoptosis and the above considerations, a variety of secondary effects on cell growth-signaling and/or survival-signaling pathways may theoretically also contribute to tumor growth inhibition following EM exposure.

### 2.2 Electromagnetic sources and tissue penetration

There are a variety of EM field delivery modes that can potentially engage with distinct tumor membrane modifications or substituents, organelles, or molecules that typically carry significant charge over a small spatial area. The latter may be susceptible to molecular force, torque, or strain upon EM field engagement. One method that has been applied is the use of alternating current (AC) electric fields with unique orthogonal array placement to vary the incident field on the tumor target, using fields in the 100–400 kHz range with field strengths of approximately 2–4 V/cm pulsed at 1 Hz. This tumor-treating field (TTF) application has been used clinically in the treatment of glioblastoma brain tumors (33). Multiple mechanisms, including pulsing of S-phase DNA/chromatin or tumor-cell microtubules, induction of tumor-specific membrane leak and stress (including nuclear and mitochondrial membranes), and other effects such as apoptosis, ROS generation, and autophagy, have been described (34, 35). With efficacy under unique conditions for which dose response (based on ***E***-field amplitude, V/cm) appears to be described (35), the dominant mechanisms remain to be determined.

Interestingly, electric field attenuation across tissue may result in unique challenges in achieving optimal and uniform TTF effects across the dimensions of a deep-seated tumor (36). Magnetic fields can be pulsed to induce electromotive forces with unique induced voltage profiles across deep tissue. This may drive movement of charge deep in the tissue and possibly with a distinct distribution of uniform induced-EMF amplitudes across a deep-seated tumor thickness. Effects resulting from such a classical application of Faraday’s law of induction using pulsed magnetic fields (PMFs) are compelling and reasonably may operate to engage charge over a fairly wide spatial area (over contiguous tumor cells in a small/growing tumor, for example) exposed to either radiofrequency EM waves or pulsed magnetic fields (13, 15, 22, 37). While the energies may be distinct in this setting, the ability to possibly tailor PMF pulse characteristics to engage with unique tumor cell properties while maintaining a deep cross-tumor EM field uniformity may be appealing in developing platforms.

### 2.3 Consideration of frequency, pulse characteristics, and time

A major area of focus in a variety of EM field delivery models is the wave frequency that is selected to affect the biological process of interest. This is commonly reported, along with the amplitude of the EM wave (in volts per meter) or primary magnetic pulse-generating device (in Tesla) if an induction approach was used. The majority of studies do not report dose-response data in terms of physical wave characteristics and biological effects. Whether low-frequency pulsed magnetic fields are used, often in milli-Tesla to Tesla range amplitudes, or EM waves delivered in tissue-penetrating radiofrequency or infrared frequencies, there interestingly appears to be a common impact on tumor cell apoptosis (increased) and cell viability (decreased), often with variable degrees of ROS generation (2–7, 19, 24). These are usually for tumor-exposure periods on the order of hours applied daily. Also missing are mechanistic data as to how EM effects are transduced in the cell to affect growth or apoptosis. Even if reported without “dose-response” data, some insight into the mechanistic initial impact of the EM wave or pulsed magnetic field on cellular elements (plasma membrane, cytoskeleton, nucleus/chromatin, mitochondrion/membrane) would be of high interest. The dose (frequency or amplitude) may be varied from there.

### 2.4 Pulse rise and shape as a unique mechanistic key to cellular engagement

**Figure 1:**
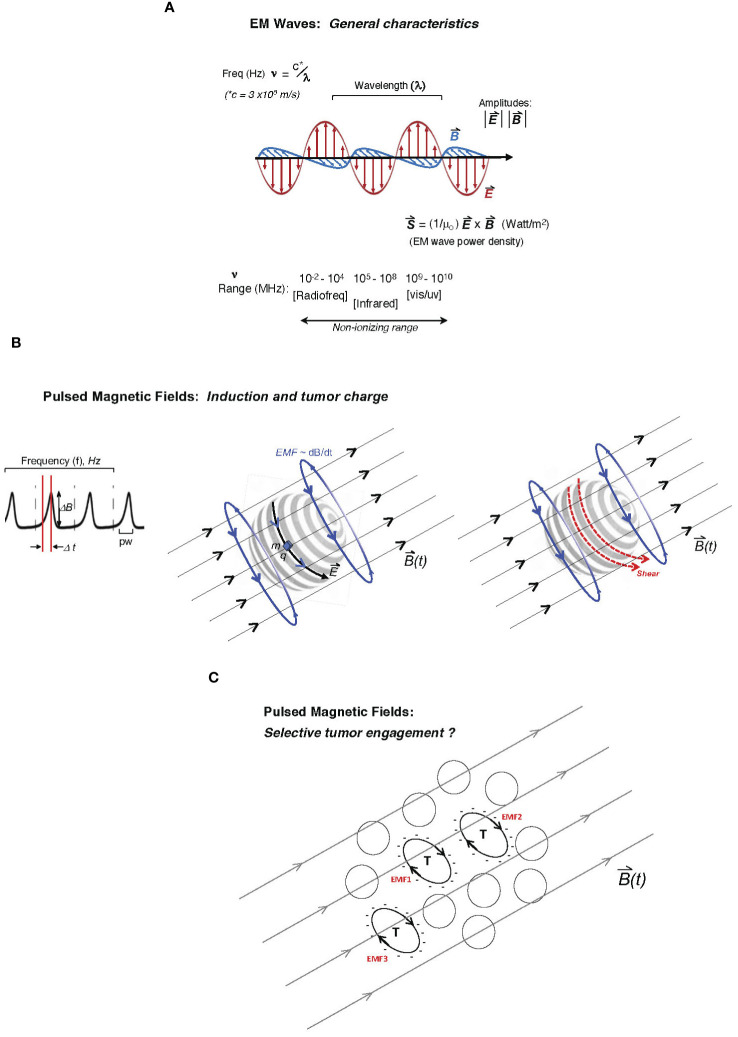
Description of two major electromagnetic (EM) platform strategies used to affect tumor behavior to date. Mechanisms of tumor cell engagement are only partially understood and discussed in the main text. In one family of EM platforms, electromagnetic waves **(A)** are delivered as traveling waves in space and characterized by frequency (ν), wavelength (λ) (with product c, the speed of light) as well as power density, and direction defined by the Poynting vector (*S*, proportional to cross-product of *E*- and *B*-fields) shown below the illustrated EM wave with orthogonal electric and magnetic vector components, oscillating at frequencies emitted typically by radiofrequency-generating equipment to affect tumor cell biological behavior or even inflammatory tissue remodeling. While attenuated by interaction with tissue, the ability of such radiation to affect deeply seated tumor cells is governed by the dielectric properties of tissue. Frequencies that may affect tumor cell viability in plated tumor-cell studies may extend beyond the radiofrequency range and to the high non-ionizing range (infrared or even vis-light range; bottom). **(B)** In pulsed delivery systems, pulsed EM wave “packets” may be delivered as pulsed electromagnetic fields (PEMFs); alternatively, a pulsed magnetic field (PMF) may be applied to generate induced electromotive forces (EMFs; voltage fields orthogonal to the pulsing external *B*-field) deep in tissue through which the time-dependent magnetic field, *B*(t), penetrates, with graphed pulse-train at left, showing the pulse profile (dB/dt as the magnitude of D*B* delivered over Dt, the pulse-width; pw), with pulses delivered at arbitrary frequency, *f* (Hz), often at ultra-low pulse driving frequencies (<300 Hz). Middle illustration shows how the induced EMF, proportional to dB/dt (change in magnetic flux), can drive motion of charge element q with mass m along induced electric field vector *E* on the surface of a hypothetical tumor cell (e.g., glycocalyx charge motion) or even a “slab” of charge on a spherical cluster of tumor cells. The same pulsed forces on the tumor glycocalyx (to which charge q and mass m are attached) may create shear on the tumor cell surface (right illustration) at molecular attachments to the membrane, creating tumor cell stress and downstream viability effects. **(C)** Theoretically, a variety of tumor cells (T) with unique cell surface charge (anionic surfaces as illustrated) in a population may be susceptible to pulsed EMFs, distinctly governed by the charged areas over which dB/dt fluxes (designated as hypothetical EMF1, EMF2, etc., in red), wherein differential intra-tumoral effects and stress are possible.

An under-reported and key variable in empirical tumor-model exposures to EM platforms is the shape of the duty cycle of the applied EM wave or pulsed/oscillating magnetic field. This parameter may be particularly critical in the transduction of tumor cellular and molecular functions in response to the EM stimulus, including metabolic or growth-related stress. For a PMF approach, the rise or slope of each pulse may critically determine the degree of mechanistic coupling to a uniquely susceptible cellular element in malignant cells. In physical terms, the rate of rise in a magnetic pulse or oscillation (i.e., its “sharpness”) is conveyed as dB/dt (13, 15, 38). The EMF induced by that particular period of rise to the maximum amplitude may be more impactful on unique tumor cellular features (e.g., abnormally expressed molecular charge on the plasma membrane, microtubular features, or the state of chromatin). Specifically, in a classical consideration of Faraday’s principle, it may be proposed that the rate of change in the magnetic flux density (dB/dt across area A) induces an EMF bounding area A, which can uniquely couple with charge-dominated tumor cell-specific elements (15, 26, 30). **Figure 1** illustrates both the classical EM wave (**Figure 1A**) and the PMF and how induced EMFs via the PMF approach may engage selectively with charged elements unique to tumor cells (single or in a contiguous colony) while sparing non-tumor cells or tissues. Such EMFs may engage with distinctly charged tumor membranes to induce molecular torque and shear forces along with cancer cell stress, with the example in **Figure 1B** describing critical pulse train characteristics such as amplitude, frequency/period, duty cycle, rise time, and pulse width (top) and how induced EMFs may drive membrane shear by engaging tumor-overexpressed molecular charge and mass distributions. While a uniquely charged tumor plasma membrane is used in the illustration, the pulsed field may disrupt any number of candidate tumor-unique structures or states, including membrane integrity, microtubule or spindle function, chromatin integrity, or the generation of ROS in parallel to apoptosis as a direct or downstream result of tumor mitochondrial disruption. These may be sensitive to varying distinct pulse characteristics (e.g., rise time), and distinct tumor “nests” may be distinctly stressed through independent EMF effects (**Figure 1C**).

The shape of a pulse delivered over the “duty cycle” is characterized by rise time as well as the pulse width (time over which the field is pulsed; **Figure 1B**), which can be far less than the period of pulse delivery or oscillatory rate (38). Therefore, a low-frequency PMF that effectively couples (e.g., with substantial mechanical response) to a unique tumor cellular feature such as a highly charged tumor glycocalyx may “oscillate” at either 10 Hz or 10 kHz; however, the duty cycle and dB/dt may be designed identically for these two frequencies (for example, dB/dt delivered as 10 μT over 20 μs in both cases), wherein the key to efficacy lies in the duty cycle rise time over 20 μs rather than the PMF frequency (10 Hz or 10 kHz) per se. Thus, relatively low pulse amplitudes may be used to affect tumor-characteristic properties so long as “dt” is very short (i.e., narrow pulse width), and thus the independent parameters of dBmax (amplitude) and “dt” (effectively, the period of the duty cycle) should be separately reported. As another example, independent of frequency, if the period of a pulsed 50 mT *B*-field can deliver pulses over a 50-μs duty cycle (i.e., rise time <50 μs), the reporting of “a PMF delivered at dB/dt = 1.0 kT/s” without information on the pulse-width parameters does not provide sufficient information. A PMF of 500 mT delivered over 500 μs could also be reported as “1.0 kT/s”, although such an exposure may be critically different than the former (possibly with markedly different biological effects), given the pulse delivery over a 10-fold narrower duty cycle in the former. Engagement of the induced EMF with sub-cellular structures, or even “confluent” cellular elements such as the tumor plasma membrane glycocalyx (e.g., over a microsphere of thousands of tumor cells), may critically depend on the very narrow pulse width, even if the *B*-field amplitude is much lower and independent of the PMF pulse-train frequency.

It is thus important to recognize that “pulsing” an EMF can have significant effects at ELFs, where the single pulse can be delivered at narrow pulse widths with relatively low energy (mT range) to cancer cell systems to perturb membrane activity. An important mechanistic consideration regarding how the latter is affected in experimental tumor systems under external ELF-EMF generation involves the perturbation of ion fluxes that maintain a relatively depolarized state in resting cancer cells, and the effects that this has in driving metabolic thermal-generating responses that further stress relatively extreme non-equilibrium thermal state between the tumor cell and its environment due to greater metabolic activity (39, 40).

### 2.5 The unique state of tumor membrane mechanical resonance

In theory, achieving classical mechanical resonance of a sub-cellular structure or even an entire tumor cell as a unique entity is intriguing. In this approach to an EM wave inducing tumor cell-specific resonance and stress, one may design a suitable “driving” frequency such that a full wavelength (oscillatory period) envelopes the tumor-cell diameter (including the richly anionic glycocalyx) or “fitting” a uniquely charged tumor organelle of interest in one cycle (41). With an EM wave as a pulsed electromagnetic field (PEMF), the uniquely charged tumor cell surface or even a unique organelle (tumor mitochondrion or S-phase nuclear chromatin domain) may retain unique characteristics that give it much greater charge than that of neighboring stromal cells. With a tumor cell as a target (diameters in a 10–30-μm range), a “driving” frequency in the THz range may achieve unique “capture” and tumor-cell resonance since that size is approximated by the incident EM wavelength (γ = c/ν, where c = 3 × 10^8^ m/s and ν is in THz range), drawing analogy to classical mechanical wave dynamics (41): testing/tuning γ in that range may achieve optimal “resonant” responses. Alternatively, one may capture a “clump” of tumor cell diameters with a confluent, shared glycocalyx “slab” at a frequency that is 10-fold lower (with γ in the 100–300-μm range). Unique resonant EMF “capture” without effects on neighboring stromal cells may be possible since the surface of many tumor cells *in vivo* may be distinguished from stromal cells by virtue of a more highly charged glycocalyx (26, 28, 30). Additionally, while stromal cells may have lesser degrees of the same anionic glycans on their physiologic glycocalyces, the quest may be to engage the glycocalyx with “driving” EMFs for even short mechanical capture periods against a mechanically distinct surround, regardless of whether one achieves full sustained mechanical resonance of the whole tumor cell or intracellular organelle under the pulsed driving force. Other variables to consider in any classical dynamics resonance approach include viscosity (gamma) or how the “spring” characteristics of surrounding tissue affect Q (quality factor) for any given tumor cell. These engineering variables (41, 42) are difficult to calculate to generate a theoretical “resonant frequency”, but one could potentially approach this empirically with sophisticated equipment.

In another independent application of resonance, it is intriguing that dedicated ELF-induced EMFs using coil-generated pulses in the <10-Hz range could be applied to 2D and 3D spheroid tumor cells to generate resonance with thermal dissipation that resembles or models according to resistor/capacitor “RC” circuit type behavior (39). This was achieved with the application of specific ELFs that could optimally drive responses that not only inhibited cancer cell growth but also generated ion fluxes (current) and dissipated heat. To maintain homeostasis, downstream mitochondrial responses (coupled and uncoupled activity with ATP production) appear to be boosted upon achieving this “thermal resonant frequency”.

### 2.6 Electroporation

Induction of EP by various modalities has been achieved under direct tumor contact with probes (pulsing range of 1 kHz–1 MHz). Cell death by irreversible electroporation (IRE) occurs by varying degrees via apoptosis and necrosis/necroptosis and follows through an ATP depletion effect likely triggered by Ca++ ion tumor-cell entry, in addition to possible electroconformational denaturation of macromolecules, resulting from the EP probe pulsing (43, 44). There are likely multiple mechanisms that drive IRE tumor cell damage. Curiously, the release of damage-associated molecular pattern (DAMP) molecules by EP correlates with tumor cell death (45). Notably, the use of non-EP approaches such as alternating electric fields or external PMFs has resulted in nanopore formation (<20-nm pores) in tumor cells (27, 46), which is associated with leak and membrane disruption (46), while hydrophilic nanopore formation induced by EP approaches has been described (43). This may promote varying degrees of Ca++ entry and depending on extracellular Ca++, release of ATP and other DAMPs, lipid peroxidation, and downstream generation of ROS as well as induction of apoptotic pathways or more severe cellular injury via mechanisms as discussed (38, 43, 44). This further implies that tumor antigen release and immunologic priming may take place in such a microenvironment. The ability to potentially achieve the same membrane Ca++ inward-leak effect by applying coils and low-frequency pulses outside the body (or applied regionally across a body section) is intriguing. This is also in appreciation of the penetrative ability of PMFs, with deep-tissue induced EMFs and noting that studies with directly applied IRE pulses employ dB/dt typically in the microsecond (e.g., <100 μs) pulse-width range in several clinical applications (43).

### 2.7 Tailoring EM field parameters to selectively induce tumor cell strain while empowering immunity

Multiple modalities considered above may drive tumor sensitivity to EM waves, with a variety of stress or cytotoxic tumor-cell response mechanisms that may vary significantly depending on the modality. Indeed, one or two modalities may critically affect common tumor-responsive elements such as the glycocalyx, altered mitochondrial membrane properties, and ROS sensitivity (13, 14, 24, 38, 41). Deciphering which physical parameter (e.g., EM wave amplitude or power, PMF pulse profile, dB/dt, and pulse frequency) may have the greatest impact on a specific tumor cell response is critically important in understanding how one can leverage a unique platform to achieve tumor membrane stress, apoptosis, cytotoxicity, or antigen release. The selectivity of “tuning” any empirically effective parameter to vulnerable tumor-specific cell or organelle biophysical characteristics may be an especially attractive feature while limiting strain or cytotoxicity to surrounding stromal or physiologic tissue. This is also in the spirit of the general paradigm presented herein, with a focus on preserving the function of tumor-associated or trafficking T cells, natural killer (NK) cells, and antigen-presenting cells (APCs). It is remarkable that in some experimental applications that expose carcinoma cells and even neoplastic myeloid/lymphoid cells (leukemic and lymphoma lines) to EMFs, bystander non-neoplastic primary immune cells (including lymphocytes) under the same exposure remain more resilient or intact from apoptosis or DNA fragmentation (47–49), while more generally in EMF exposed whole neoplasms, the activation and expansion of anti-tumor immune effector cells appears to be the rule (49).

Focusing on immune cells as the latter substituents to preserve and empower, an EM platform delivering the appropriate “dominant” parameter discovered from prior testing (e.g., tumor ROS generation under a unique low-frequency PMF pulse train with micro- or nanosecond pulse widths) may promote the anti-tumor function(s) of such cells without destroying them. This may ideally promote tumor antigen release and cross-presentation in a cytotoxic T cell-rich environment. Achieving this may spare immune cells and regional lymphatics participating in primary anti-tumor immunization from excessive stress or death that ensues from parallel chemotherapy or ionizing radiotherapy approaches that have traditionally been seen as “ideal” to potentiate a tumor vaccine response or an augmented cytotoxic T-cell response using antibodies to key co-inhibitory targets (e.g., anti-PD1 or anti-CTLA4) (50–52). One may thus envision preservation or promotion of the natural substrates for an optimal anti-tumor cytotoxic response with greater anti-tumor “signal-1” (tumor antigen presentation) potential as a result of the novel tumor-strain/apoptotic and tumor-cytotoxic conditions induced by the EM platform.

## 3 Immunotherapy limitations and barriers

In parallel to the EM platform, the following are discussed: a) usual and novel barriers to optimal cytotoxic T-cell responses to the challenging heterogeneous and unstable carcinoma immunologic microenvironment and (b) how EM delivery platforms may facilitate augmented cytotoxic T-cell responses within the endogenous TME or under integrated anti-cancer immunotherapeutic strategies. These considerations are introduced in keeping with the goal of the paradigm proposal herein: to communicate the importance as well as insights into considering the integration of EM platforms with immunotherapeutic approaches in the treatment of cancer. The topics in this section introduce immunologic barriers that may be favorably modulated by the incorporation of EM platforms in potential future cancer therapeutic strategies.

### 3.1 Physical and immunologic barriers: opportunities to “leverage” anti-cancer immunity

1. Physical properties of the carcinoma membrane glycocalyx. It is well known that a variety of cancers express unique glycan modifications of tumor cell-surface proteins and lipids. A common modification is the upregulation of glycosaminoglycan (GAG) polymers, attached as sulfated glycans (heparan sulfate and chondroitin sulfate), or secreted into the immediate surround of the tumor cell as sulfated GAGs or (non-sulfated) hyaluronan chains (26, 53). As a biophysical property distinct from the normal epithelial surround, such modifications endow tumor cells with substantial anionic surface charge. Moreover, sialic acid (Sia) monomer modifications of (typically heavy) tumor mucinous glycoproteins (54), which are also anionic at physiologic pH, contribute to what may be collectively considered a thick anionic glycocalyx “slab” over confluent carcinoma cell surfaces. This contributes not only to shielding functions that physically block access of cross-reactive T cells to tumor-immunologic antigens on the tumor surface but also to cell–cell “repulsion” that facilitates tumor spread and invasion (26, 55).
2. Molecular “shielding” and immune repression. The tumor cell surface can also serve specifically as an immunologic barrier. An example involves the overexpression of tumor Sia and repressive Siglec receptor-mediated signaling on NK or T cells attempting to engage with the tumor cell surface (56, 57). The physical inability to make deeper contact with tumor surface antigens also imposes a kinetic inhibition in the ability of antigen-sensitized CD4 or CD8 cytotoxic T lymphocyte (CTL) cells to activate in response to shielded cognate antigens. With a glycocalyx layer rich in Sia, immunogenic epitopes may become “blocked” or shielded by the presence of heavy terminal glycan Sia modifications (28, 56, 58).
3. Immunologic “desert” and bystander T cells. One kinetic argument regarding both naïve T cells and tumor antigen-sensitized cytotoxic T cells and NK cells in the TME is the inability to approach or “remain near” tumor antigen sources or tumor-cell nests in the tumor mass. Such nests are often “islands” among a stromal cell surround, and the additional glycocalyx shielding may isolate potential anti-tumor T cells and NK cells from tumor cell nests, despite chemokine and cytokine signals that may typically promote migration and improved proximity. Such stromal cells, including cancer-associated fibroblasts and remodeling endothelium, can promote tumor a pre-invasive phenotype. This occurs because of the ability of such stromal cells to promote pro-metastatic conditions and a TME that suppresses anti-tumor immunity, although the behavior of stromal cells can also vary with TME spatial distribution (59).
4. Extracellular matrix (ECM) as a barrier. The tumor ECM is often rich in dense networks of highly enriched secreted molecules that can further shield tumor cells from immune cells in the TME. These networks are often rich in the same anionic molecules (e.g., glycosaminoglycans on secreted proteoglycan core proteins) that promote attachment of growth factors via basic amino acid-rich domains in the form of “banks” for advancing tumor cells or remodeling vasculature (60, 61). Enzymes such as heparinase or hyaluronidase released by such cells may also mobilize such factors in the promotion of tumor growth, while the tumor ECM barrier and interstitium expand the barrier to surrounding immunologic cells.
5. Tumor blood flow is a barrier to the delivery of anti-tumor “cargo”. The tortuous and leaky vascular supply of tumors, which expands through the action of a variety of pro-angiogenic tumor growth factors (e.g., VEGF mitogen family, FGFs, and PDGF) (62), is relatively inefficient in extending capillary beds throughout tumor nests, often reaching an extreme in central hypoxic tumor zones that become necrotic. This limits access to anti-tumor agents (molecular or cell therapies) to the tumor mass as well as endogenous colonization by circulating anti-tumor T cells or NK cells. Tumor vascular proliferation under tumor VEGF stimulation is not only tortuous but also leaky, with a net effect of increasing intra-tumor interstitial pressure (63), which further limits the ability of systemic or tumor-peripheral immune cells (including naïve or sensitized cytotoxic T cells) to access the inner depths of the tumor (63, 64).
6. Tumor lymphatic microenvironment: barriers to immune cell traffic. Tumor lymphatic vasculature may serve as a means for naïve and sensitized T cells to access regions of the tumor that may be susceptible to immunologic attack and release of tumor antigens. The latter, including antigens from metastatic or dying tumor cells (possibly boosted by cytotoxic therapies), may flow to regional lymph nodes via draining lymphatic vessels to regional lymph nodes, or even tertiary lymphoid structures (TLSs) along lung bronchovasculature, providing antigen substrate for APC uptake and cross-presentation with immune activation and proliferation of anti-tumor cytotoxic T cells (65). Tumor dendritic cells may also traffic in the same direction, carrying tumor antigen for presentation to draining nodes. Unfortunately, elevated tumor interstitial pressure (as per the previous section discussion) limits the depth to which lymphatic egress may carry antigens from deeper portions of the heterogeneous tumor mass.

**Figure 2:**
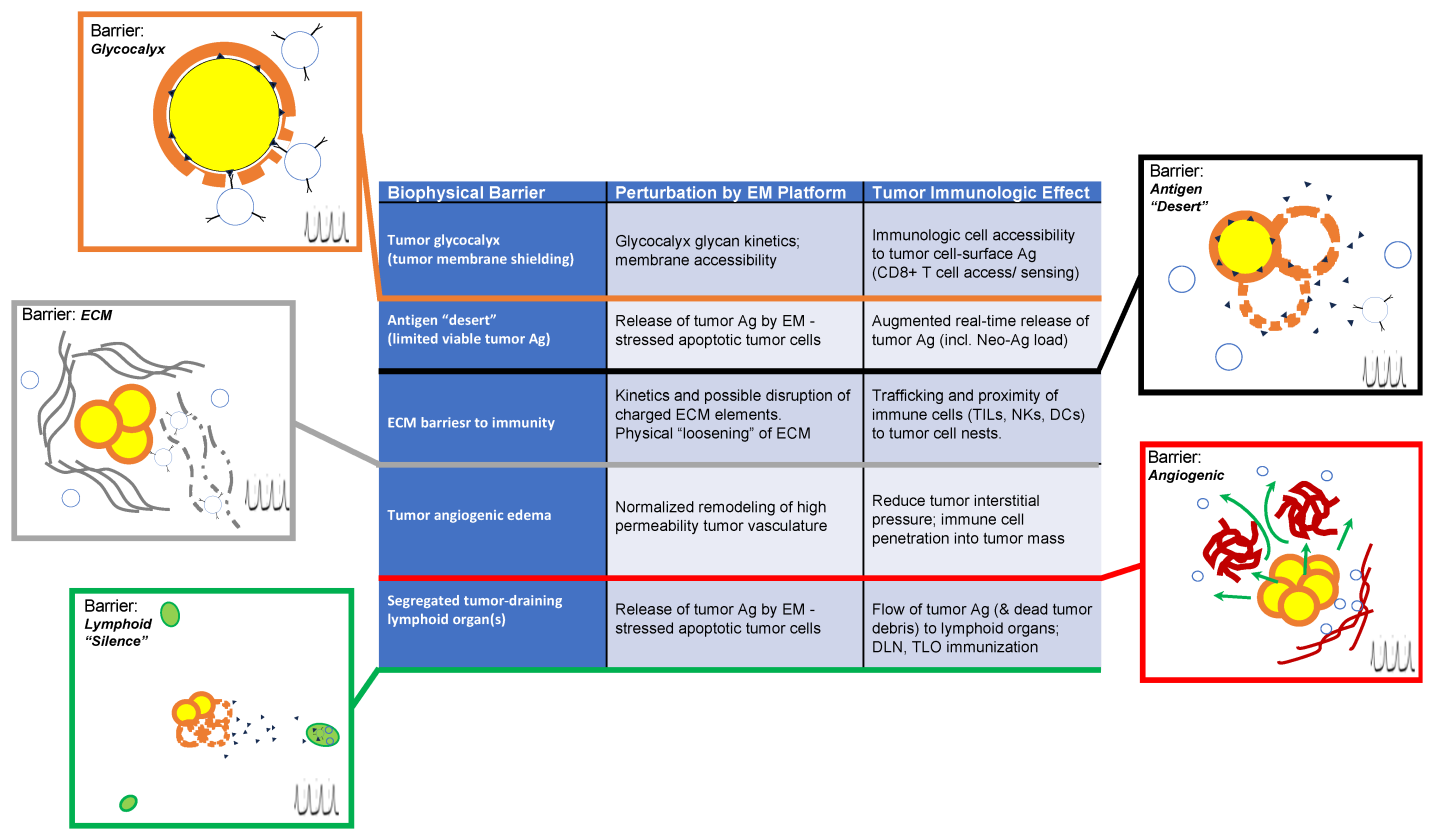
Illustrated summary of biophysical barriers to potential augmented anti-tumor immunologic priming through electromagnetic (EM) perturbations of the tumor microenvironment. Each barrier is indicated in the center (left column) along with descriptions of physical perturbations that may be achieved through EM platform exposure (middle column) and potential specific immunologic events (right column) that may be augmented through EM platform exposures that can (directly or indirectly) modify or eliminate barrier condition(s). For pictorial attachments corresponding to each barrier, the upper left side of each illustration states the barrier and illustrates the nature of the barrier under tumor-pathologic conditions, while lower right (indicated by a pulsed-field symbol) illustrates modified conditions that correspond to post-EM exposure effects on the respective barrier. This illustrates how immunologic priming or “access” of immune cells (whether naïve or sensitized) may be augmented by the specific effect(s) of EM exposure on the specific barrier under consideration.

**Figure 2** summarizes a variety of physical and immunologic barriers to anti-tumor therapeutic approaches, with a focus on how EM platforms may directly or indirectly augment “access” to anti-tumor cellular immunity through biophysical EM effector mechanisms that address each barrier (summarized in the table at center).

## 4 Electromagnetic platforms may generate the “substrates” for improved anti-cancer immunity

**Figure 3:**
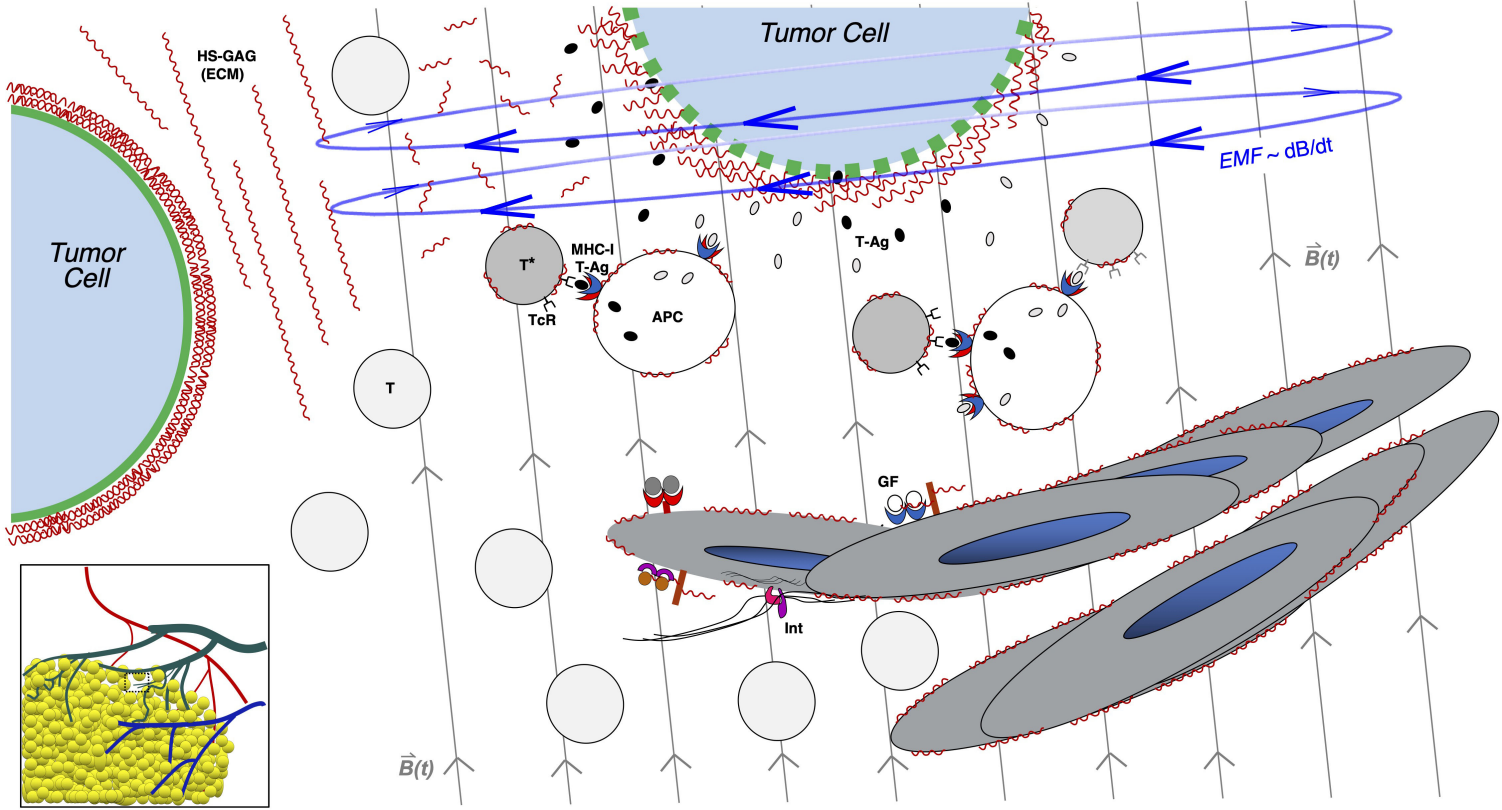
Induction of non-thermal and non-ionizing electromagnetic force in the tumor microenvironment: potential consequences of glycocalyx disruption. Tumor cells (top and to left) are illustrated in a growing region on the edge of carcinoma, with figure as a zoomed inset in lower-left cartoon of vascularized tumor mass. Tumor cells are characterized by thick surface glycocalyces, as illustrated by dense regions of wavy lines (in red) surrounding the plasma membrane in green, as in tumor cells at baseline (left side) while surrounded by extracellular matrix (ECM) polymers rich in heparan sulfate glycosaminoglycan (HS-GAG) chains, represented by extracellular red wavy lines to right of cell. Also shown nearby is a vascular remodeling region depicting lymphangiogenic responses to tumor vascular growth factors (GFs; such as VEGF family members) as well as integrins (Int), growing into center. Nearby immune cells such as naïve T cells (T) or activated T cells (T*) and antigen-presenting cells (APCs) may be sensitized against tumor antigens (T-Ag) in this tumor microenvironment. As an EM platform is applied in this case as an external pulsed magnetic field depicted by vertical light gray lines across center and right side of figure; *B*(*t*), the induced EMF shown in blue, with circular field lines orthogonal to the pulsed magnetic field (and proportional to dB/dt), engages with the charge-rich tumor glycocalyx (tumor cell at top, within field), distorting it and inducing leak on the tumor membrane (gaps in green membrane of cell). This may induce tumor cell stress and release of tumor antigens (T-Ag; small gray and black ovals) and promote CD8+ T-cell sensitization and activation by nearby APCs, which may cross-present T-Ag on MHC-I (illustrated by blue/red APC receptor). This illustrates one way by which anti-tumor immunity may be augmented by an EM platform of pulsed magnetic fields applied to the tumor microenvironment.

Electromagnetic induction has the potential to “loosen” barriers and engage levers to augment acquired T-cell immunity and checkpoint drivers while avoiding the ablation of effector immune cells in the same environment. Importantly, chemotherapy and ionizing radiation have the detrimental potential to eliminate or impair functional anti-tumor immune cells in the TME, including immunologic cells that are critical in mediating regional anti-tumor immunity (51, 52). These may include naïve T cells, innate immune cells, or cells that have acquired specific immunity, including CD8+ cytotoxic T cells as well as CD4+ effectors sensitized to tumor antigens. It is intriguing to consider how TME immune barriers may be overcome or eliminated under unique EM platforms:

1. EM engagement with physical properties of the tumor glycocalyx. With unique upregulation of anionic sulfated glycans such as Sia or charged polymer (e.g., sulfated GAG or hyaluronan) modifications that project into the tumor glycocalyx in a variety of carcinomas, the potential to induce tumor membrane shear stress through pulsed EMF engagement with a “slab” of glycocalyx exists. In a macroscopic sense, a slab of glycocalyx covering a continuous layer of multiple cancer cells may potentially be driven into pulsed motion, creating shear on the tumor membrane via strain on protein and lipid attachments of the glycocalyx to the membrane (30). This can induce membrane leak and downstream cell stress (27, 30). This must be examined methodically for distinct tumors, and if PMFs are used, a key may be to achieve sufficient dB/dt to induce capture and force (slab mass × acceleration), rather than frequency or even amplitude of the field per se. The induction intensity (dB/dt) may well be more critical than the field maximum amplitude (*B*_max_) in this setting (13, 15, 30). Moreover, if this affects tumor glycocalyx with relative selectivity, without engaging glycan surfaces of stromal or immune cells (with markedly lower surface anionic charge densities), then this differential may allow optimum conditions for EM platform-induced (and relatively tumor-selective) apoptosis, antigen release, and sensing by TME immune cells.
2a. EM induction of molecular force and torque: Consequences on tumor cell strain and viability. In tumor cells rich in proteins and lipids modified heavily by charged glycans, forces by pulsed *B*-field induced EMFs may induce motion of heavily charged glycosylated “head” regions of molecules, with a significant lever arm about the base of molecular attachment to the plasma membrane. This torque has the potential to deform the membrane with forces in the pico-Newton range (66, 67). Glycosidic bonds are generally strong and potentially less likely to “give”, thus transmitting forces into marked deformation and strain at the membrane-attached bases of proteoglycan core proteins, glycoproteins, or glycolipids; see references for examples of orders of magnitude pertaining to relative free energies of dissociation (68–70). This may result in either a leak near points of torque (i.e., “weak points”) or mechanical transduction of neighboring channel proteins to affect ionic or small-molecule transport (71). Empirically, the effects have also been shown to induce outward leak of plasma proteins, including proteases as well as the formation of nanopores with diameters in the 5–20-nm range: when electromagnetic pulse widths are substantially under the μs range, pore sizes appear to become substantially smaller (27, 46, 72).
2b. Stress, leak, and cell death by *any* electroporation modality in tumor cells. Ideally, one can achieve “selective electroporation” as a result of engaging a unique EM platform (e.g., PMF-induced EMFs) with tumor glycocalyx to ultimately drive leak and cell stress, for example, via tumor membrane nanopore formation. The same platforms may remain relatively inert in neighboring non-tumor cells as a result of distinct (non- or low-charged) glycocalyces and the absence of overexpressed Sia or GAGs (26, 30, 73). Interestingly, the induction of aminocyanine modifications on tumor membranes followed by light exposure was sufficient to induce rapid pore formation via “molecular jackhammers” through vibronic action on tumor plasma membranes, and immediate cell death. This is a novel two-step method for the transduction of vis-light waves to dramatic cytolytic effects in tumor monolayers and mouse tumor models (74). The effect is appealing for carcinoma applications and possibly novel clinical translation.
3. Release of tumor-antigen targets in an immune “desert”. Could the immediate or delayed effects of selective tumor-cell electroporation and/or downstream apoptosis as a result of EM-induced cancer cell stress result in the release of tumor (neo)antigens into the TME while leaving bystander naïve or sensitized anti-tumor T cells relatively unharmed? The destruction of immune cells, including T and B cells in the TME as well as secondary lymphoid organs (lymph nodes) or TLS domains on nearby bronchovascular bundles, can be the unfortunate result of chemo- or ionizing radiotherapy, resulting in a loss in the opportunity for sensing of tumor antigen(s) as a “side effect” of classical tumor ablative modalities (50–52). This may create an immunologic “desert” within heterogeneous TME regions, with tumor-antigen targets as a lost opportunity in a setting of immunologic cell death or stress. Alternatively, immunity may be harnessed broadly in a modality that is potentially more selective in inducing tumor-specific stress, including apoptosis/necroptosis while “sparing” immunologic viability in a setting of EM field exposure.
4. The carcinoma ECM is also rich in charged glycans and primed for mobilizing immunity. It may be envisioned that engagement of EM platforms with charged molecular components of a tumor glycocalyx (e.g., glycocalyx-resonant EM waves or PMFs inducing EMFs that drive tumor molecular torque) may also engage susceptible poly-anionic matrix molecules that may not be tightly attached to tumor plasma membranes. Rather, secreted GAG or Sia decorated mucin-rich tumor cell products surrounding tumor nests or modifying basement membranes as glycan-rich ECM barriers may be vulnerable to EM waves or magnetic pulsing to disrupt or “loosen” a kinetic barrier to immunologic traffic (75, 76), including both tumor antigen sensitized or naïve T cells, gaining access to tumor-cell products, which also include apoptotic or tumor dead-cell fragments. Reductions in matrix heparan sulfate and decorin have also been noted following the IRE of intact lungs in animal models (76). This may potentially remodel the peri-tumor ECM in a way that improves anatomic points of entry for effector immune cells, but one can also consider whether this could change the “exit potential” for tumor cells from nests, considering potential consequences on local invasion. This may be empirically weighed against a greater exposure substrate for anti-tumor immunity that may be driven by this potential interplay. One can question how this may be visualized or measured. When surviving sensitized T cells are physically distanced by significant interstitium or ECM, then EM field effects on the matrix itself can facilitate T-cell penetration or narrow the bridge length remaining for CTLs to make signal 1 (MHC/antigen) contacts on tumor surfaces. In this light, EM exposures may also facilitate bi-specific T-cell engager (BiTE) compounds [tarlatamab (77)], which engage a bridge between CD3 on the T cell and DLL3 on small cell lung carcinoma cells.
**Figure 3** illustrates how an EM stimulus that engages unique properties of the tumor plasma membrane (and even ECM) may promote mechanisms resulting in anti-tumor immune activation. Some of the above “substrates” for acquired immunologic activation and expansion are shown in the stromal and vascular microenvironment of the tumor, with the cartoon highlighting examples of glycocalyx sensitivity and engagement, possibly inert or physiologic molecular players, and secondary steps that promote immune activation against a stressed tumor cell within the penumbra of an EM induction field.
5. Promoting transfer of immune-cellular or anti-tumor therapeutics to glycan-”vulnerable” cancer cells. The focus here is promoting greater “proximity” between potential/naïve or sensitized immune effector cells [e.g., dendritic cells (DCs), T cells, and NK cells] and/or therapeutic agents and tumor nests. Tumor vascular proliferation, primarily stimulated under common VEGF-A splice variants (e.g., VEGF-165) in tumor endothelium, is not only tortuous but also leaky (hence original term vascular permeability factor/VPF for VEGF-A) (78). It promotes a net effect of increasing intra-tumor interstitial pressure, which further limits the ability of systemic or tumor-peripheral immune cells to gain access to viable tumors while accelerating necrosis in other parts of the tumor (79). The latter may contain some antigen for sensing, but the absence of viable tumors (and immune cells) in such regions may hamper quality immune responses or outward traffic of newly sensitized T cells from the toxic environment (80). Interestingly, there are reports of pulsed EM radiofrequency fields generated on patient- or pet-oriented rings (e.g., Assisi or Beamer coils) used in anti-inflammatory and wound-healing contexts in veterinary practice for promoting improved or “normalized” blood flow (e.g., in granulation tissue) to facilitate wound recovery or improved inflammatory healing rates (e.g., post-traumatic musculoskeletal injury) in large animals (14, 25). Whether this “straightens” or normalizes vasculature in tumors to create a similar response in tumor blood flow as that of anti-VEGF therapy (81), following the discovery of VEGF-A induced “tortuous” tumor angiogenesis, and improves/lowers tumor interstitial pressure is unclear. Nevertheless, the concept of “normalizing” tumor vasculature through EM platform exposure while promoting anti-tumor immunity remains appealing as a strategy to promote a functional and “penetrant” tumor vasculature as a result of unique remodeling (via unknown mechanisms) while reducing tumor interstitial pressure.
6. Promoting lymphatic recruitment of secondary and tertiary lymphoid centers. It is appealing to envision that tumor antigens that can activate germinal centers in secondary lymphoid organs could flow (as metastatic or dying tumor cells or even free antigens) to regional lymph nodes via draining lymphatic vessels. These include secondary or even tertiary lymphoid structures (along lung bronchovasculature, for example), providing antigen substrate for APC cross-presentation with immune activation and proliferation of anti-tumor cytotoxic T cells in the lymphoid organ(s) (65). Of course, the same conduit can carry live tumor cells with the danger of lymph node metastasis. Activated DCs (as master APCs) can also traffic in the same lymphatic beds, carrying processed tumor antigens, with downstream presentation events in draining lymph nodes. This may further include tertiary lymphoid structures (e.g., TLS domains along peri-tumor and lobar bronchovascular bundles in the setting of lung cancer). In any EM platform that can potentially impact/lessen tumor interstitial pressure through vascular remodeling or “normalization”, it is possible that such lymphatic traffic can drain even deeper portions of the tumor while potentially boosting antigen release into the draining “pool” in the form of greater apoptotic tumor cells or free antigen-rich molecular products following pulsing or electroporation, for example (49).

**Table 1** summarizes a variety of cellular mechanisms by which EM platforms may augment anti-tumor immunity via induction in a non-ionizing and non-thermal manner. Some of these may occur through indirect effects of tumor cell strain or death in the microenvironment of naïve and/or sensitized T cells, antigen-presenting cells, NK cells, and vascular endothelial cells, among others.

**Table 1:** Mechanisms by which non-ionizing, non-thermal EM platforms may augment anti-tumor immunity.

Function	Effector cells impacted	Possible mechanism(s)
CD8+ T-cell antigen priming	Naïve and effector T cells	Release of antigen from EM-driven tumor apoptosis or necrosis. Nanopore formation by reversible or irreversible electroporation
Tumor antigen sensing	APCs (mainly cDC1 DCs)	Augmented antigen cross-presentation in setting of unstable (pulse-vibrated) Sia or GAG chains (intact glycans regulate presentation spatially/temporally)
“Open” or decompress ECM for effector migration/kinetics	APCs and T cells (naïve and effector)	EM induced inhibition in matrix density (temporal), facilitated immunologic cell penetration to tumor mass
Inhibit glycan-mediated immune repression	NK cells, T cells. Also DCs “in *trans*” (matrix) or “in *cis*”	Perturbation or downregulation in Sia (considered a “resistance” response) on immune cells may inhibit Siglec-mediated immunosuppressive signaling
Possible deep regional tumor immune-cell access	Endothelial cells in the TME; circulating T cells	Vascular remodeling; possible “normalizing” effects on tumor vessels, reducing tumor interstitial pressure

EM, electromagnetic; APCs, antigen-presenting cells; Sia, sialic acid; GAG, glycosaminoglycan; ECM, extracellular matrix; NK, natural killer; DCs, dendritic cells; TME, tumor microenvironment.

## 5 Discussion

### 5.1 A novel integrated pathway to boost anti-cancer immunity

The above observations and insights suggest the optimal integration of an EM carcinoma cell stress modality with immunologic sensing and tumor-cell contact by well-primed T cells. This includes contact with non-viable tumor cells and/or antigen products as debris evolving from EM-induced apoptotic and necroptotic tumor cells in the TME. In the setting of EM wave exposures induced either “at a distance” by external PMF induction or penetrating low-frequency radiofrequency EM waves or by a tumor-proximal probe source (e.g., irreversible electroporation source deployed to the surface of the tumor), the combination of tumor cell stress and antigen release is carried out while aiming to leave immunologic cell bystanders relatively intact and ready to prime in response to changes in the microenvironment (49, 82). Emerging studies in animal models are demonstrating anti-tumor immunologic preservation (and boost) of non-thermal electroporative strategies, for example, in direct comparison to that of radiofrequency thermal ablative approaches in the same model (83). If cell-surface properties of DCs, T cells, NK cells, and stromal cells are unique and remain non-vulnerable to detrimental effects of EM stimuli (e.g., transduced by discrete or continuous glycocalyces of tumor cells), then one may achieve scenarios where tumor nests are stressed, even to a lethal extent that promotes tumor antigen release via apoptosis and necroptosis (84, 85), while minimizing stress on the immune effectors. The latter has been shown to expand in the post-treatment TME of a variety of IRE-treated tumors in animals and humans (86).

The “sparing” and effective activation of effector T cells that may be achieved under EM platform-driven tumor-stress modalities in the TME may not be the case in most common chemotherapy or ionizing radiotherapy platforms, even when paired with immunotherapy. Further, while common current immunotherapy strategies for carcinoma are immune checkpoint inhibitor (ICI) based and operate optimally (and without antigen specificity) in response to target antigen/MHC–T-cell “signal one” events, exposure to tumor-stress focused EM platforms may augment signal one response by bystander naïve T cells (or antigen-driven expansion of tumor-sensitized T cells) in the same TME in real time. This may facilitate exposure of tumor-generated immune substrates for acquired anti-tumor immunity and memory T-cell generation despite a constantly evolving carcinoma antigen landscape. A challenge in the evolution of tumor surface “contact” PEF platforms in particular is maintaining an even exposure of any PEF over the full tumor landscape due to the electric heterogeneity of tumor tissue (87), leaving the potential for tumor recurrence in differentially exposed regions with incomplete IRE-directed ablation (86). Possibly, the use of locally delivered PMFs may expose the full thickness of tumors to induced EMFs (dB/dt) more evenly due to uniform *B*-field penetration and thus more uniform release of the variety of antigens in the evolving heterogeneous tumor landscape.

As an example, in lung cancer as well as metastatic tumors to the lung, the use of bronchoscopic IRE is being investigated for augmentation of the unique effects that this EM platform has on inducing a local effector tumor immunophenotype, characterized by increased DCs, effector T cells, inhibition in T-suppressor (Treg subset) cells, and augmented regional TLS activation, while IRE has been more generally shown to spare stromal and vascular cells within the TME (75, 86, 88). This specific PEF platform, which is growing in very early clinical application, delivers electric pulses directly to the center of the lung tumor with the bronchoscope-deployed IRE probe, wherein energy is the greatest in a central IRE ablation zone, while a gradient likely exists wherein tumor immune cells are differentially sensitized to the radially outward attenuating *E*-field delivered with sufficient energy to induce tumor-cell stress and/or death without appreciable thermal effects on surrounding vascular/stromal tissues (75, 88). The tumor cell stress and release of antigens in this context may boost cross-presentation to CD8+ T cells with the induction of cytolytic effector cells in distinct regions. One could conjecture that regional intra-tumor or cross-tumor delivery of a PMF in such a “probe-delivered” manner could also non-thermally and uniformly alter distinct clonal tumor populations through the full tumor thickness to release “regional” antigens across the heterogeneous carcinoma. Boosting this with ICI or other novel approaches while the local immune system is preserved may provide a unique variation on this theme.

Finally, studies are needed to understand how PEFs (e.g., delivered via IRE) or PMFs may directly affect immune cells, possibly via interactions with their respective glycocalyces: the latter may be substantially less dense than that of tumor cells but may have unique properties under PEF or PMF stimulation at distinct frequencies. For example, one can demonstrate dose-dependent PMF-induced selective cellular protease leak of glioblastoma and neuroblastoma tumor cells, while primary bone marrow DCs exposed to the identical platform remain resistant to leak (30). Further studies are needed to assess how the exposure affects the behavior and performance of the DCs upon antigen exposure or cross-presentation to T cells. The boost in the latter due to indirect tumor-antigen exposure and promoting effects of EM platforms via direct non-thermal tumor strain and lethality seems plausible as one (if not the) key mechanism, but one cannot yet rule out direct effects of the EM platform on the immunologic cells (APC function, T-cell activation, and others).

### 5.2 Biological consequences of tumor EM-platform resistance?

The scenario above may further operate as cancer “Achilles’ heel”. This is not solely due to more optimal anti-tumor immune “leverage” during simultaneous cancer cell stress or membrane impairment (which is intended to be relatively selective under the EM platform) but also because the development of tumor resistance to the EM platforms may have serious consequences on the tumor’s success in tissue invasion, spread, and even survival. How may this be? As an example, a tumor’s “addiction” to a unique glycocalyx, even if this varies for distinct tumor cells, may be converted to a “death sentence” for the tumor under an EM tumor-stress platform.

For at least a large subset of tumors expressing heavy membrane Sia modifications (e.g., as poly-sialic acid or Sia-modified glycoproteins and glycolipids/gangliosides) on the tumor-cell glycocalyx (26, 28, 89, 90), a natural “resistance” response to escape EM-induced tumor stress may evolve through the tumor’s attempt to reduce Sia expression, thus reducing the tumor’s dense glycan anionic charge layer. However, a high density of tumor glycocalyx Sia (particularly polysialic acid polymers) has been shown to be important for physical tumor cell–cell repulsion at a tissue-invading tumor front, including repulsion from immune effector cells (55, 90–92). It may also mediate key binding of chemokines and cytokines that affect tumor cell migration and behavior (92). Heavy glycocalyx Sia also induces immunologic shielding from anti-tumor immune cells through the activation of repressive Sia-binding Siglecs on immune-cell surfaces (for example, NK-surface Siglec 7-, 9-, or NKG2D-mediated repression of NK cells by engagement with heavy Sia on tumor glycocalyx) (56, 57). Siglec-mediated repression by tumor-surface Sia expression is also a theme for other anti-tumor immune cells in the TME. In yet another scenario of potential EM-platform tumor resistance, remodeling attempts by tumor cells to reduce the expression of anionic GAGs such as hyaluronan or sulfated HS chains (which can “dominate” the glycocalyx mass in a variety of carcinomas) to escape or survive EM-induced tumor-cell stress may lead to tumor growth inhibition due to loss of major growth factor co-receptor functions by such GAGs (55, 61, 93). A reduction in “banks” of growth factors bound to secreted GAGs in the tumor ECM in this scenario may also result in reduced tumor invasion through basement membranes and tissue barriers (61). Therefore, the loss of repulsion, immune escape, or growth potential resulting from tumor attempts to reduce key anionic tumor glycocalyx constituents as an EM-platform resistance mechanism may result in a “checkmate” situation for the glycan-addicted tumor cell that must hover between EM-platform vulnerability and loss of invasive and immune-shielding potential.

It is further appealing to consider from a thermodynamic standpoint the need for tumor cells to maintain a relatively high surface area/volume ratio to optimally exchange heat with the environment as a result of their greater resting metabolism and heat generation (94). In unique EM platforms with ELF-EMF induction, a perturbation that induces greater metabolic stress and heat (to maintain homeostasis in ion flux perturbations) has impacts on limiting tumor cell growth and division. It is interesting to conjecture that if the tumor cell attempts to become “resistant” to these effects by changing membrane shape (and/or motility in parallel), the impact further limiting heat dissipation may become lethal. Again, in this example, attempted resistance to an EM platform may become a viability “trap” for exposed tumor cells. Indeed, tumors that have progressed to advanced stages may be especially vulnerable given insights into greater heat dissipation at higher stages (95).

**Figure 4:**
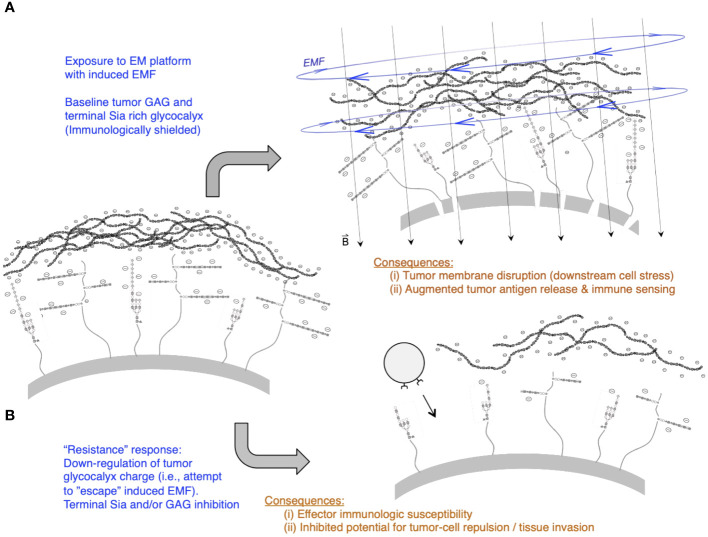
Tumor glycocalyx and temporal dynamics of applying a pulsed magnetic field (PMF) EM platform. **(A)** Short-term effects of the PMF include possible lateral forces as well as molecular torque-induced stress and membrane leak resulting from the induced EMF that pulses at the driving frequency. Note perforations or “pores” along tumor (upper right scenario) resulting from movement of a dense anionic glycocalyx “slab” that consists of overexpressed hyaluronan (top layer of charged polymers) as well as other heavily expressed glycans such as GAG chains (e.g., heparan sulfate) and complex branched Sia capped glycoproteins and glycolipids/gangliosides in the glycocalyx “canopy” that decorates core proteins and lipids attached at the tumor plasma membrane base. An additional consequence of this short-term membrane-disrupting effect is tumor cell stress, downstream apoptosis, and tumor antigen release contributing as substrates for immunologic sensing. **(B)** Longer-term potential downregulation of charged glycans by tumor cells that may “escape” PMF-induced electromotive forces (EMFs): a key consequence of this may be loss of charged glycans (lower right scenario) that are essential for tumor cell repulsion, matrix migration, and repressive signals upon contact with nearby immune cells (e.g., Sia loss leading to a reduction in repressive Siglec-mediated signaling on NK cells and T cells).

**Figure 4** illustrates scenarios of i) EM platform exposure that disrupts the tumor membrane due to glycocalyx “slab” movement and molecular torque that may occur on an EM-strained and disrupted tumor membrane, inducing leak and downstream mechanisms that promote tumor cell stress and potentially tumor cell lethality (as a mechanism for augmented tumor antigen exposure in the TME). ii) Under temporal “resistance” by an exposed tumor cell, escape from these effects is possible, but with consequences of heightened immunologic susceptibility due to the downregulation of glycans that typically serve to repress incoming anti-tumor NK cells or T cells through a variety of glycan–receptor (e.g., Siglec and other) immunologic cell-inhibitory pathways.

### 5.3 Future gauging of tumor sensitivity in real-time EM-immunologic platforms

An ultimate last step would be pairing any form of immunotherapy (even current-day ICI strategies) with an EM wave platform that can be specifically “tuned” for optimal tumor membrane stress from a fresh biopsy specimen from the tumor with its accompanying stroma. Effectively, with any real-time tumor specimen, one could determine a ratio of cytotoxic “leak” of proteases (or inward leak of a tracking molecule such as propidium iodide) by tumor cells versus that of stromal cells separated from the same specimen (30). In classical dynamic scenarios of mechanical resonance (42), this is essentially the resonance “quality factor” of the tumor-glycocalyx (as an EM-wave susceptible system) relative to that of non-tumor cells in the immediate environment. If one envisions true resonance by an EM wave, this could be reported as such (i.e., a “gamma” value for the microscopic “co-culture” system under EM exposure); however, the more common physical comparison or ratio of importance (if resonance is not achieved) could simply be that of tumor-cell leak due to an optimal dB/dt leak-inducing pulsed magnetic field (numerator) relative to that of non-tumor stromal cells that ideally demonstrate minimal leak under the same stimulus (denominator). Essentially, one could envision that with a patient’s tumor tissue biopsy in hand, empirical analyses of the effects of a range of EM platform exposures on tumor membrane leak and stress may guide the application of a specific “responsive” EM platform to the patient as part of an “EM adjuvant” therapeutic platform.

More challenging but promising future technology and innovation platforms may allow for external EM input to reflect or capture onto nano-antenna data reception and analysis systems. It may even be possible to induce unique EM platform-mediated stress through “tuning” of induction dB/dt or amplitude, frequency, and time secondary measures (low-frequency PMF platform, for example) and simultaneously detect anionic glycocalyx perturbation or molecular torque-mediated membrane responses as an effective tumor “glycocalyx response biopsy” as the system is tuned in real time, possibly even without the need for tissue. Dissecting “spectral” type read-outs during the EM delivery stimulus may tease tumor glycocalyx-specific responses from that of distinct non-tumor stromal responses by referencing emission patterns of reference normal-tissue standards.

## Data availability statement

The original contributions presented in the study are included in the article/supplementary material. Further inquiries can be directed to the corresponding author.

## Author contributions

MF: Writing – review & editing, Writing – original draft, Supervision, Resources, Methodology, Investigation, Funding acquisition, Conceptualization.
